# Validation of cytoplasmic-to-nuclear ratio of survivin as an indicator of improved prognosis in breast cancer

**DOI:** 10.1186/1471-2407-10-639

**Published:** 2010-11-23

**Authors:** Elton Rexhepaj, Karin Jirstrom, Darran P O'Connor, Sallyann L O'Brien, Goran Landberg, Michael J Duffy, Donal J Brennan, William M Gallagher

**Affiliations:** 1 UCD School of Biomolecular and Biomedical Science, UCD Conway Institute, University College Dublin, Belfield, Dublin 4, Ireland; 2 Center for Molecular Pathology, Department of Laboratory Medicine, Lund University, Skåne University Hospital, Malmö, Sweden; 3 UCD School of Medicine and Medical Science, UCD Conway Institute, University College Dublin, Belfield, Dublin 4, Ireland; 4 Department of Pathology and Laboratory Medicine, St. Vincent's University Hospital, Elm Park, Dublin 4, Ireland

## Abstract

**Background:**

Conflicting data exist regarding the prognostic and predictive impact of survivin (BIRC5) in breast cancer. We previously reported survivin cytoplasmic-to-nuclear ratio (CNR) as an independent prognostic indicator in breast cancer. Here, we validate survivin CNR in a separate and extended cohort. Furthermore, we present new data suggesting that a low CNR may predict outcome in tamoxifen-treated patients.

**Methods:**

Survin expression was assessed using immunhistochemistry on a breast cancer tissue microarray (TMA) containing 512 tumours. Whole slide digital images were captured using an Aperio XT scanner. Automated image analysis was used to identify tumour from stroma and then to quantify tumour-specific nuclear and cytoplasmic survivin. A decision tree model selected using a 10-fold cross-validation approach was used to identify prognostic subgroups based on nuclear and cytoplasmic survivin expression.

**Results:**

Following optimisation of the staining procedure, it was possible to evaluate survivin protein expression in 70.1% (n = 359) of the 512 tumours represented on the TMA. Decision tree analysis predicted that nuclear, as opposed to cytoplasmic, survivin was the most important determinant of overall survival (OS) and breast cancer-specific survival (BCSS). The decision tree model confirmed CNR of 5 as the optimum threshold for survival analysis. Univariate analysis demonstrated an association between a high CNR (>5) and a prolonged BCSS (HR 0.49, 95% CI 0.29-0.81, p = 0.006). Multivariate analysis revealed a high CNR (>5) was an independent predictor of BCSS (HR 0.47, 95% CI 0.27-0.82, p = 0.008). An increased CNR was associated with ER positive (p = 0.045), low grade (p = 0.007), Ki-67 (p = 0.001) and Her2 (p = 0.026) negative tumours. Finally, a high CNR was an independent predictor of OS in tamoxifen-treated ER-positive patients (HR 0.44, 95% CI 0.23-0.87, p = 0.018).

**Conclusion:**

Using the same threshold as our previous study, we have validated survivin CNR as a marker of good prognosis in breast cancer in a large independent cohort. These findings provide robust evidence of the importance of survivin CNR as a breast cancer biomarker, and its potential to predict outcome in tamoxifen-treated patients.

## Background

Personalised medicine, whereby individuals receive tailored therapeutic regimens based on individual patient and tumour characteristics, is now felt to be an achievable goal. Effective implementation of personalised cancer therapeutic regimes, however, depends upon the successful identification and translation of informative biomarkers to aid clinical decision-making [1]. The role of immunohistochemistry (IHC) within this arena is most likely to involve predictive biomarker development, as highlighted by the classical success of both estrogen receptor (ER) and Her2 in breast cancer, which predict response to tamoxifen and trastuzumab, respectively.

Survivin (encoded by the gene, BIRC5), a member of the inhibitor of apoptosis protein family, is a multifunctional protein implicated in a number of cellular processes including apoptosis, mitosis and angiogenesis [2]. Survivin has been proposed as a promising tumour biomarker mainly due to work using serial analysis of gene expression (SAGE), which revealed that survivin was the fourth most highly expressed transcript in a number of common cancers, but was rarely present in normal terminally-differentiated tissues [3]. Multiple studies in several different tumour types have investigated the prognostic value of survivin [2]; however, many IHC-based studies have been hampered by a failure to reach a consensus regarding how survivin staining should be interpreted. Principally, discordance has focused on whether examination of the cytoplasmic fraction, nuclear fraction or both provide more useful information. Using IHC or subcellular fractionation, two pools of survivin have been located (nuclear and cytoplasmic). These different pools are immunochemically and functionally different and are independently modulated during cell cycle progression [4].

Although it exhibits a high degree of tumour-specific expression [3,5], and is one of the 16 cancer-related genes represented in the Oncotype DX assay [6], the role of survivin as a breast cancer biomarker has remained the subject of much debate (1). Previous studies of survivin expression measured using qRT-PCR or IHC in primary breast cancer have reported that it is either prognostically irrelevant [7-9], or associated with improved [10] or adverse outcome [11-13]. Such discordant results could perhaps be explained by the fact that these studies did not account for subcellular localisation of survivin. Survivin is often simultaneously expressed in both the cytoplasm and the nucleus, making manual analysis of IHC difficult; however, the introduction of digital imaging devices and computer-assisted image analysis has provided a major advance towards quantitative description of IHC signals [14].

We previously applied automated quantitative algorithms to analyse survivin IHC data and demonstrated that increased expression of nuclear, as opposed to cytoplasmic, survivin was associated with a decreased overall survival (OS) and breast cancer-specific survival (BCSS) [15]. A high cytoplasmic-to-nuclear ratio (CNR) was associated with low grade, hormone receptor positivity and improved OS and BCSS. Multivariate analysis demonstrated that a high CNR (>5) was an independent predictor of a prolonged survival [15].

This was the first study to examine the relationship between survivin CNR and outcome and is consistent with the hypothesis that nuclear and cytoplasmic survivin fractions have different biological functions [4]. These earlier findings suggested that nuclear survivin is a marker of poor prognosis in breast cancer and that automated analysis can be used to quantify nuclear survivin. However, given the large number of conflicting studies previously reported [2], a validation study is required. The aim of the current study was to use advanced pattern recognition algorithms to validate survivin CNR as a prognostic biomarker in an independent breast cancer dataset.

## Methods

### Patients

This study included 512 consecutive patients with primary invasive breast cancer treated and diagnosed at Malmö University Hospital between 1^st ^January 1988 and 31^st ^December 1992 [16-18]. The median age at diagnosis was 65 (range 27-96) years and median follow-up time to first breast cancer event was 128 months (0-207). Information regarding the date of death was obtained from the regional cause-of-death registries for all patients. Complete treatment data were available for 379 (76%) patients, 160 of whom had received adjuvant tamoxifen. Twenty-three patients received adjuvant chemotherapy. Two hundred patients received no adjuvant systemic treatment. Ethical permission was obtained from the Local Ethics Committee at Lund University (Dnr 613/02), whereby informed consent was deemed not to be required other than by the opt-out method.

### Tissue microarray construction

TMAs were constructed using two 0.6 mm cores taken from areas representative of invasive cancer and mounted in a recipient block using a manual arraying device (MTA-1, Beecher Inc, WI, USA) as previously described [19]. TMA blocks were stored in Dept. of Pathology in Malmö University Hospital, Sweden. TMA sections were cut immediately prior to staining.

### Immunohistochemistry

Sections (4 μm) were dried, deparaffinised and rehydrated through descending concentrations of ethanol. Heat-mediated antigen retrieval was performed using microwave treatment for 2 × 5 min in a citrate buffer (pH 6.0), before being processed either in the Ventana Benchmark system (Ventana Medical Systems Inc, AZ) using pre-diluted antibodies to ER (Anti-ER, clone 6F11), progesterone receptor (PR) (Anti-PgR, clone 16) and Her2 (Pathway CB-USA, 760-2694) or in the Dako Techmate 500 system (Dako, Glostrup, Denmark) for Ki-67 (1:200, M7240; Dako) and survivin (1:50, D-8 Santa Cruz, CA). ER, PR and Ki-67 were quantified using a commercially available automated nuclear algorithm (*IHC-MARK*; OncoMark Limited, Ireland), as previously described [20]. ER, PR and Ki-67 negativity was defined as < 10% positively stained nuclei. Her2 staining was evaluated according to a standard protocol (HercepTest) and scored as 4 intensities (i.e. negative, weak, moderate and strong), namely 0-3+; these scores were divided into two groups, with negative to weak (0-2+) Her2 expression (Her2^-^) and strong (3+) overexpression (Her2^+^).

### Image acquisition, management and analysis

The Aperio ScanScope XT Slide Scanner (Aperio Technologies, Vista, CA) system was used to capture whole slide digital images with a 20× objective. Digital images were managed using Spectrum (Aperio). Tumour and stromal elements were identified using Genie (Aperio) pattern recognition software and a quantitative scoring model for both nuclear and cytoplasmic survivin was developed using the positive pixel count algorithm (Aperio).

### Statistical analysis

Spearman's Rho correlation was used to estimate the relationship between duplicate cores from individual tumours. Differences in distribution of clinical data and tumour characteristics between samples with a high and low survivin nuclear autoscore (SNAS) and CNR were evaluated using the χ^2 ^test. Kaplan-Meier analysis and the log rank test were used to illustrate differences between OS and BCSS according to survivin expression. Cox regression proportional hazards models were used to estimate the relationship to OS and BCSS of survivin, patient age, lymph node status, tumour grade, Her2, PR, and ER status in the patient cohort. For decision tree analysis, all patients were randomly divided into 10 subsets. A decision tree model was selected using a 10-fold cross-validation approach. Ten consecutive decision tree models were independently constructed using the SNAS and CNR continuous output from 9 subsets. Prognostic accuracy of each decision tree model was tested using the remaining set of patients, with the model displaying the highest accuracy being selected as the optimal model for the dataset. All calculations were performed using SPSS version 12.0 (SPSS Inc, Chicago, IL). A p-value < 0.05 was considered statistically significant.

## Results

### Automated quantification of survivin protein expression in breast cancer

The specificity of the anti-survivin antibody was confirmed in our previous study [15]. Following optimisation of the staining procedure, it was possible to evaluate survivin protein expression in 70.1% (n = 359) of the tumours represented on the TMA. To evaluate our study for any potential selection bias, baseline clinicopathological characteristics from both the evaluated ("survivin known") cohort (n = 359) and the unevaluated ("survivin unknown") cohort (n = 153) are presented in Table 1. This illustrates that the clinicopathological characteristics were well matched in both evaluated and unevaluated cohorts.

**Table 1:** Clinical and tumour characteristics of evaluated cohort (*n *= 359) and entire cohort on TMA (*n *= 512) stratified according to survivin nuclear autoscore (SNAS) and cytoplasmic-to-nuclear ratio (CNR) of survivin protein expression.

	Entire Cohort			Evaluated Cohort			Evaluated Cohort		
	
	(n = 512)			(n = 359)			(n = 359)		
	
	Known	Unknown	p value	SNAS < 4.26	SNAS > 4.26	p value	CNR < 5	CNR > 5	p value
						
	(n = 359)	(n = 153)		(n = 201)	(n = 158)		(n = 198)	(n = 161)	
**Age (Years)**									
**Median (Range)**									

< median (25-65)	53(49.1)	175(48.7)	0.520	98(48.8)	77(48.7)	0.541	99(50)	76(47.2)	0.598

> median (65-96)	55(50.9)	184(51.3)		103(51.2)	81(51.3)		99(50)	85(52.8)	

**Tumour Size**									

0 - 20 mm	255(71.6)	84(79.2)	0.119	148(73.6)	107(69)	0.340	134 (68.7)	121(75.2)	0.180

> 21 mm	101(28.4)	22(20.8)		53(26.4)	48(31)		61(31.3)	40(24.8)	

Unknown	3			3			3		

**Nodal status**									

N0	203(63.6)	66(67.3)	0.502	110(62.1)	93(65.5)	0.537	118(65.9)	85(60.1)	0.337

N1+	116(36.4)	32(32.7)		67(37.9)	49(34.5)		61(34.1)	55(39.2)	

Unknown	40			19			19		

**NHG**									

I&II	237(66.2)	73(67.6)	0.788	145(72.5)	92(58.2)	0.005	119(60.1)	118(73.7)	0.007

III	121(33.8)	35(32.4)		55(27.5)	66(41.8)		79(39.9)	42(26.2)	

Unknown	1								

**ER status**									

ER <10%	81(22.9)	25(23.8)	0.843	40(20.2)	41(26.3)	0.176	53(27.1)	28(17.7)	0.038

ER >10%	273(77.1)	80(76.2)		158(79.8)	115(73.7)		143(72.9)	130(82.3)	

Unknown	5			2			2		

**PR Status**									

PR <10%	151(42.7)	50(47.6)	0.368	82(41.4)	69(44.2)	0.595	89 (45.4)	62(39.2)	0.243

PR >10%	203(57.3)	55(52.4)		116(58.6)	87(55.8)		107(54.6)	96(60.8)	

Unknown	5			2			2		

**Ki-67**									

< 10%	128(27.8)	35(37.2)	0.926	82(43.9)	46(30.3)	0.010	55(29.2)	73(48.3)	<0.001

> 10%	211(62.2)	59(62.8)		105(56.1)	106(69.7)		133(70.7)	78(51.6)	

Unknown	20			10			10		

**Her2 IHC**									

Normal/weak (0 - 2^+^)	285(80.5)	81(77.1)	0.451	166(83.9)	119(76.3)	0.075	149(76.1)	136(86.1)	0.018

Overexpressed (3^+^)	69(19.5)	24(22.9)		32(16.1)	37(23.7)		47(23.9)	22(13.9)	

Unknown	5			2			2		

Abbreviations: SNAS = Survivin Nuclear Autoscore, CNR = Cytoplasmic to Nuclear Ratio, N0 = node negative, N1+ = node positive, NHG = Nottingham Harris Grade, ER = estrogen receptor, PR = progesterone receptor, Her2 IHC = Her 2 immunohistochemistry

**Figure 1:**
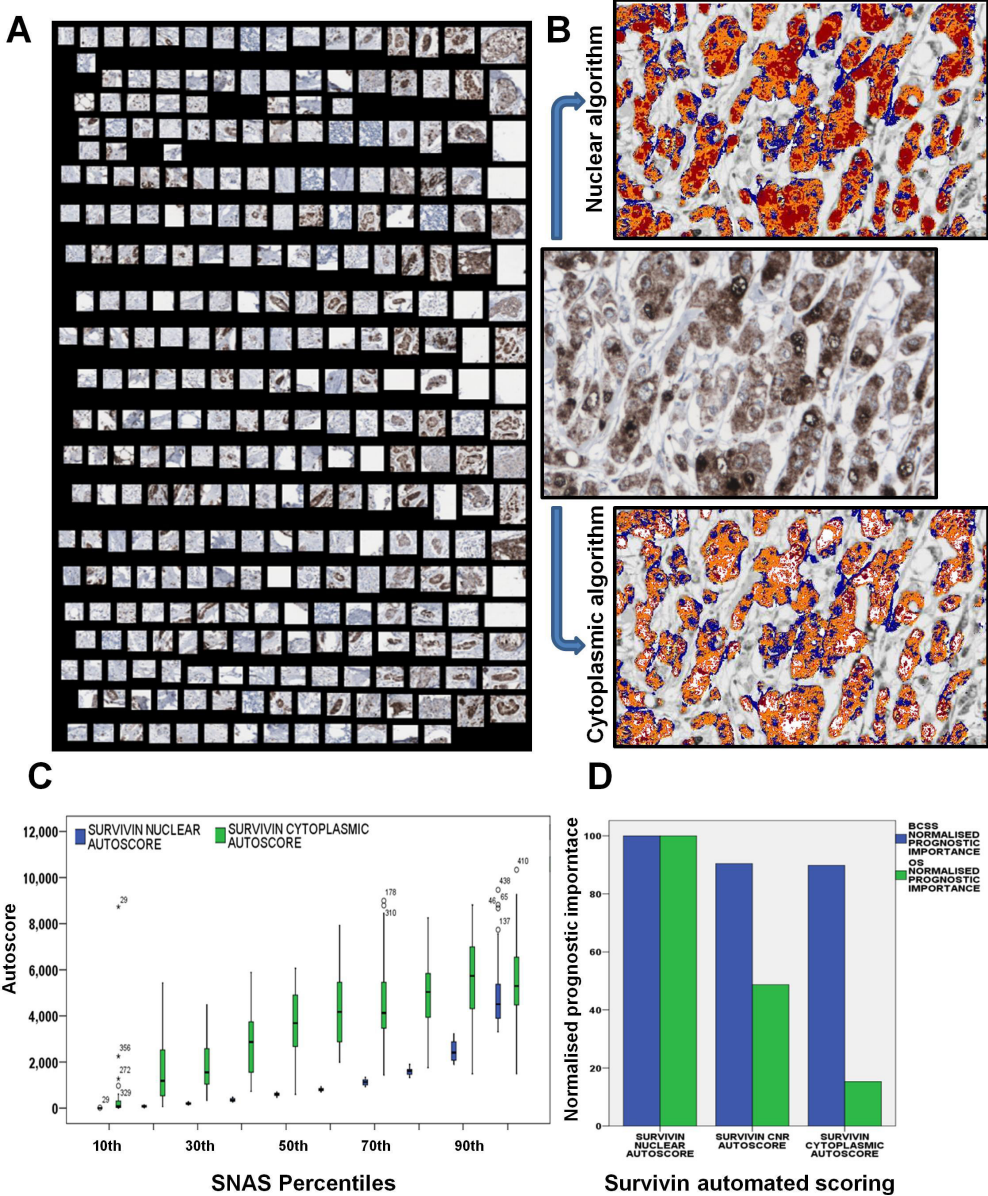
**Automated quantification of survivin immunohistochemistry**. **(A) **Representative areas from tumour positive, tumour negative, tumour stroma, tumour stroma cells and slide background were selected as a training set to develop a pattern recognition algorithm. **(B) **Examples of IHC (20X) showing nuclear and cytoplasmic expression of survivin and mark-up images with the corresponding output from the automated quantification approach. The algorithm maps survivin expression by labelling nuclear expression in red and cytoplasmic expression in orange and yellow. **(C) **Box-plot demonstrating relationship between survivin nuclear and cytoplasmic autoscores. **(D) **Bar chart showing normalised prognostic importance of SNAS, CNR and survivin cytoplasmic autoscore, as defined by the decision tree classification and regression trees.

In order to develop an accurate quantitative automated model of survivin expression, a pattern recognition algorithm developed in Genie (Aperio) was used to identify tumour from stroma and slide background. This algorithm was trained using five prospectively selected patterns - 1) tumour positive, 2) tumour negative, 3) stromal fibroblast and extracellular matrix, 4) lymphocytes and 5) slide background (Figure 1A). Following manual annotation of the training set, the five patterns were provided as an input for the pattern recognition algorithm in order to select the optimum approach. Performance of the pattern recognition algorithm was then tested in 20 randomly selected tissue cores (validation set). Comparison of automated quantification to manual annotation at pixel level resolution within the validation set revealed that the automated approach had an accuracy of 87.3%. Additional file 1 illustrates the training and output from the pattern recognition algorithm.

The positive pixel count (Aperio) was then applied to develop a quantitative scoring model for tumour-specific nuclear and cytoplasmic survivin expression. A pseudo-colour "mark-up" image was generated for each core as the algorithm output (Figure 1B). The accuracy of the algorithm was examined in high power fields (Figure 1B) and was deemed acceptable by a histopathologist.

The algorithm was used to calculate total survivin intensity, as well as cytoplasmic and nuclear intensity for each core. One of the issues to consider when examining staining intensity alone is that even a small fraction of staining artifact can significantly alter intensity values. Therefore, to reduce noise secondary to staining artifact SNAS (which combines the product of percentage positive nuclei and nuclear intensity) was proposed as an alternative automated scoring model for evaluating nuclear survivin expression. A combined percentage and intensity autoscore was calculated for both nuclear and cytoplasmic survivin expression, as previously described [15]. A strong correlation was evident between duplicate cores from individual tumours for nuclear percentage (Spearman's Rho = 0.350; p < 0.001), nuclear intensity (Spearman's Rho = 0.479; p < 0.001), cytoplasmic percentage (Spearman's Rho = 0.620; p < 0.001) and cytoplasmic intensity (Spearman's Rho = 0.619; p < 0.001), indicating a homogeneous pattern of expression. As tumours were arrayed in duplicate, the maximum value for each tumour was used for further analysis.

### Nuclear, as opposed to cytoplasmic, survivin correlates with outcome in breast cancer

We previously described a relationship between the CNR of survivin and patient outcome [15]; however, the relationship between nuclear and cytoplasmic survivin expression within individual tumours was not investigated. Comparison of nuclear and cytoplasmic autoscores from individual tumours revealed a good correlation between maximum nuclear and cytoplasmic autoscore (Spearman's Rho = 0.753; p < 0.001), suggesting that nuclear and cytoplasmic expression co-exist. In an attempt to further evaluate the relationship between nuclear and cytoplasmic survivin expression, patients were divided into groups based on SNAS centiles (Figure 1C). This revealed a close relationship between nuclear and cytoplasmic survivin. When nuclear survivin was low, cytoplasmic survivin was also low; likewise, when nuclear survivin was high, cytoplasmic survivin was also high. However, a divergence between the two measures in the mid range values suggests that a ratio such as the CNR may be useful.

Decision tree analysis was then used to examine the prognostic role of nuclear and cytoplamic survivin expression. Using a 10-fold cross-validation approach, the normalised importance of SNAS, survivin cytoplasmic autoscore and CNR was computed in relation to BCSS and OS (Figure 1D). SNAS was consistently the most prognostic marker for OS and BCSS followed by CNR, again suggesting an important prognostic role for nuclear survivin.

### Survivin nuclear autoscore is an independent predictor of outcome

**Figure 2:**
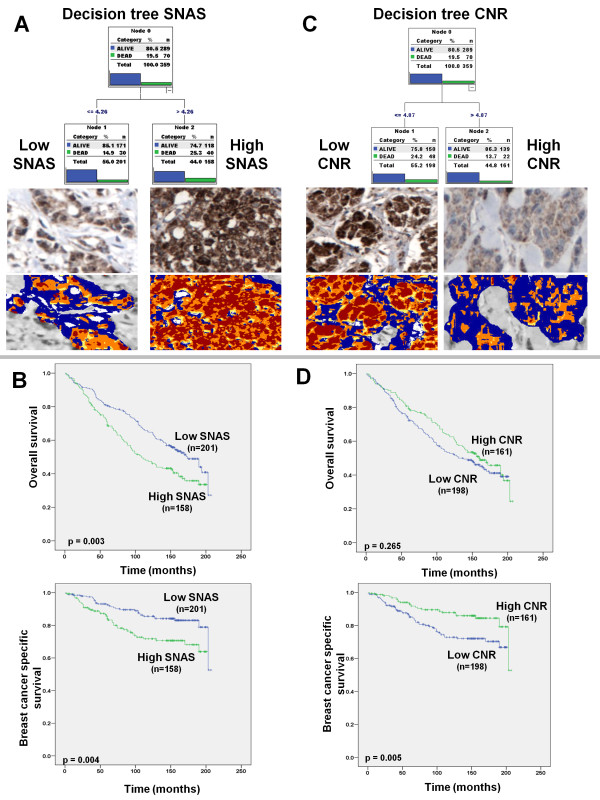
**Survivin CNR and SNAS correlate with patient outcome**. **(A) **Schematic view of the decision tree prognostic classification model based on the continuous automated quantification of SNAS. Examples of breast cancer specimens with low and high SNAS, as defined by the decision tree prognostic model, are also shown. **(B) **Kaplan-Meier estimation of OS and BCSS comparing patient with high and low SNAS, as defined by the decision tree prognostic model. **(C) **Schematic view of the prognostic classification model based on the continuous automated quantification of survivin CNR and examples of breast cancer specimens with low and high CNR, as defined by the decision tree prognostic model. **(D) **Kaplan-Meier estimation of OS and BCSS comparing patient with high and low CNR, as defined by the decision tree prognostic model.

In our initial study, we reported no link between survivin nuclear or cytoplasmic intensity and survival; however, an increased SNAS was associated with a reduced BCSS [15]. Application of the same SNAS threshold (8) in this cohort demonstrated no association between SNAS and either BCSS (p = 0.123) or OS (p = 0.298). However, univariate cox regression analysis of SNAS as a continuous variable revealed an association between SNAS and a decreased BCSS (HR 1.028, 95% CI 1.001-1.056; p = 0.044). A decision tree model was thus employed to identify the optimal threshold for SNAS in this cohort (Figure 2A) and predicted 4.26 as an optimal cut-off for automated SNAS (Additional file 2).

Kaplan-Meier analysis comparing low (n = 158) and high (n = 201) SNAS, as defined by decision tree analysis, revealed that a high SNAS was associated with decreased OS (p = 0.003) and BCSS (p = 0.004) (Figure 2B). Cox univariate analysis demonstrated that SNAS was a predictor of a reduced BCSS (HR 2.05, 95% CI 1.27-3.31, p < 0.001) and OS (HR 1.51, 95% CI 1.13-2.01, p = 0.005). Multivariate Cox regression analysis controlling nodal status, grade, ER status, PR status, Her2 status, tumour size and age revealed that SNAS was an independent predictor of OS (HR 1.68, 95% CI 1.22-2.32, p = 0.002) and BCSS (HR 1.84, 95% CI 1.10-3.08, p = 0.02), along with lymph node status, grade and age (Table 2). The relationship between SNAS and other clinicopathological variables was also examined (Table 1). A high SNAS was associated with high grade (p = 0.005) and Ki-67 positivity (p = 0.010). No relationship was evident between SNAS and patient age, nodal status, hormone receptor expression or Her2 status.

**Table 2:** Cox univariate and multivariate analysis of recurrence free and overall survival in entire cohort

	BCSS			OS		
	
	HR (95%CI)		*p*	RR (95%CI)		*p*
	
All patients (n = 359)						
**SNAS**	***Univariate***			***Univariate***		

***low***	1.00			1.00		

***high***	2.05(1.27 - 3.31*)		< 0.001	1.51(1.13 - 2.01)		0.005

**SNAS**	Multivariate*			Multivariate*		

***low***	1.00			1.00		

***high***	1.84(1.10 - 3.08)		0.020	1.68(1.22 - 2.32)		0.002

**All patients (n = 359)**						

**CNR**	***Univariate***			***Univariate***		

***low***	1.00			1.00		

***high***	0.49(0.29 - 0.81)		0.006	0.85(0.64 - 1.13)		0.267

**CNR**	Multivariate*			Multivariate*		

***low***	1.00			1.00		

***high***	0.47(0.27 - 0.82)		0.008	0.73(0.53 - 1.02)		0.067

* Multivariate analysis included adjustment for grade, age, nodal status, ER status, PR status

Her2 and tumour size

### Cytoplasmic-to-nuclear ratio of survivin is an independent predictor of disease-specific survival

Having demonstrated a significant relationship between SNAS and survival, the CNR of survivin protein expression was examined. The initial study used a non-supervised approach to identify a CNR of 5 to divide patients into groups with low and high CNR [15]. Decision tree models were again used to identify an optimal threshold for CNR. This model predicted a cut-off of 4.87 (Figure 2C) as the optimal threshold for CNR, which correlated well with the value used in the previous study [15]. There was an excellent correlation between low CNR and high CNR defined using a threshold of 4.87 or 5 (R = 0.983, p < 0.001). Patients were thus divided into groups of low and high CNR using 5 as a threshold on the automated continuous scores (Additional file 2).

Forty-nine percent (n = 161) of tumours had a CNR greater than 5. Kaplan-Meier analysis of OS and BCSS revealed that a survivin CNR of < = 5 was associated with a reduced BCSS (p = 0.005) but had no effect on OS (p = 0.187) (Figure 2D). Univariate Cox regression analysis (Table 2) confirmed the relationship between CNR and an improved BCSS (HR 0.49, 95% CI 0.29-0.81, p = 0.006). Multivariate analysis controlling for age, tumour size, nodal status, grade, ER, PR and Her2 status revealed that an increased survivin CNR was a predictor of a prolonged BCSS (HR 0.47, 95% CI 0.27-0.82, p = 0.008), along with tumour grade and lymph node status (Table 2). The relationship between survivin CNR and other clinicopathological variables was also examined. An increased survivin CNR was associated with ER-positive (p = 0.045), low grade (p = 0.007), Ki-67-negative (p = 0.001) and Her2-negative (p = 0.026) tumours (Table 1).

### Nuclear survivin expression predicts outcome in ER-positive tumours

**Figure 3:**
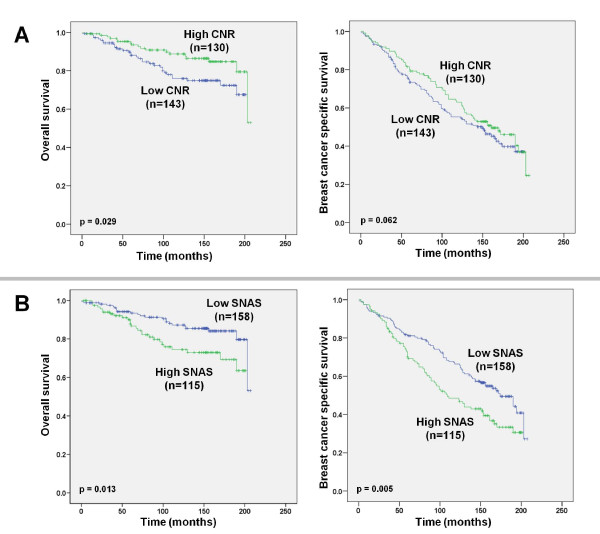
**Increased nuclear survivin predicts outcome in ER-positive patients**. **(A) **Kaplan-Meier estimation of OS and BCSS comparing ER-positive patients with high and low CNR, as defined by the decision tree prognostic model. **(B) **Kaplan-Meier estimation of OS and BCSS comparing ER-positive patients with high and low SNAS, as defined by the decision tree prognostic model.

Given the previously described relationship between increased CNR and ER positivity, we proceeded to examine the relationship between nuclear survivin and outcome in ER-positive patients. Nuclear survivin was measured as an increased SNAS or a low CNR. A low CNR (<5) was associated with a decreased BCSS (p = 0.029) in ER-positive patients (n = 273) (Figure 3A), an effect that was not evident in ER-negative (n = 81) patients (p = 0.062). A low CNR (<5) was also associated with a trend towards a decreased OS (p = 0.062) in ER-positive patients (Figure 3A). A high SNAS was also associated with a reduced BCSS (p = 0.005) and OS (p = 0.013) (Figure 3B) in ER-positive patients and a reduced BCSS (p = 0.036), but not OS (p = 0.136) in ER-negative patients.

**Figure 4:**
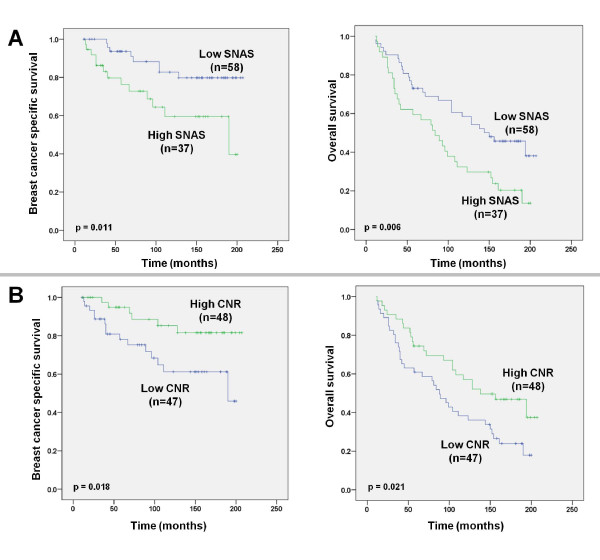
**Increased nuclear survivin predicts outcome in ER-positive patients**. Kaplan-Meier estimation of OS and BCSS in ER-positive, tamoxifen treated patients stratified according to **(A) **CNR and **(B) **SNAS.

Having demonstrated that nuclear survivin predicts outcome in ER-positive patients, we proceeded to examine its effect on ER-positive patients who received tamoxifen (n = 125). This revealed that a high SNAS was associated with a reduced OS (p = 0.006) and BCSS (p = 0.013) (Figure 4A) in tamoxifen-treated ER-positive patients. Likewise, a low CNR was associated with a reduced OS (p = 0.021) and BCSS (p = 0.018) in ER-positive patients who received tamoxifen (Figure 4B). Multivariate Cox regression analysis demonstrated that neither SNAS (HR 1.87, 95% CI 0.71-4.88, p = 0.194) nor CNR (HR 0.41, 95% CI 0.15-1.13, p = 0.087) were independent predictors of BCSS in tamoxifen-treated patients; however, a low CNR was an independent predictor of OS in tamoxifen-treated patients (HR 0.44, 95% CI 0.23-0.87, p = 0.018), after controlling for age, tumour size, nodal status, grade, PR and Her2 status (Table 3).

**Table 3:** Cox regression analysis of recurrence free- and overall survival in ER positive patients

	BCSS		OS	
	
ER Positive (n = 285)	HR (95% CI)	*p*	HR (95% CI)	*p*
**SNAS**	***Univariate***		***Univariate***	
***low***	1		1	
	
***high***	2.01(1.14 - 3.54)	0.014	1.57(1.14 - 2.16)	0.005
	
**SNAS**	***Multivariate***		***Multivariate***	
	
***low***	1		1	
	
***high***	1.38(0.71 - 2.66)	0.335	1.36(0.90 - 2.06)	*0.140*
**SNAS**	***Multivariate + treatment adjusted***		***Multivariate + treatment adjusted***	

***low***	1		1	
	
***high***	1.87(0.71 - 4.88)	*0.194*	1.92(0.98 - 3.78)	0.055
	
**CNR**	***Univariate***		***Univariate***	
	
***low***	1		1	
	
***high***	0.52(0.29 - 0.94)	0.032	0.85(0.61 - 1.13)	0.320
	
**CNR**	***Multivariate***		***Multivariate***	
	
***low***	1		1	
	
***high***	0.805(0.52 - 1.22)	0.315	0.54(0.27 - 1.10)	0.094
	
**CNR**	***Multivariate + treatment adjusted***		***Multivariate + treatment adjusted***	
	
***low***	1		1	
	
***high***	0.41(0.15 - 1.13)	0.087	0.44(0.23 - 0.87)	0.018

* Multivariate analysis included adjustment for grade, age, nodal status, PR status, Her2 and tumour size

## Discussion

High-throughput screening methodologies, particularly genomic and transcriptomic profiling have revolutionised the scientific approach to highly complex diseases such as breast cancer [21]. The potential now exists to gather increasingly complex biomedical and molecular data to develop personalised therapeutic regimens. Personalised medicine requires the discovery and application of unambiguous prognostic, predictive and pharmacodynamic biomarkers to inform therapeutic decisions [1,22]. One of the disappointing aspects of the post-genomic era is that while a plethora of putative biomarkers have undergone preliminary clinical evaluations, only a small minority have received regulatory approval for clinical use. This attrition rate has been attributed to the lack of validation studies. Here, we describe the validation of survivin CNR in a large consecutive breast cancer cohort.

Using automated image analysis and decision tree analysis, we demonstrated that nuclear, as opposed to cytoplasmic, survivin is a major predictor of outcome in breast cancer. Increased SNAS was associated with a reduced BCSS and OS (Figure 2), while an increased CNR was associated with an improved BCSS (Figure 2), confirming the relationship between nuclear survivin and poor outcome. Multivariate analysis confirmed that both measures were independent predictors of outcome (Table 2), thus validating our previous findings associating nuclear survivin with a poor outcome [15]. Although the threshold used to dichotomise patients based on high versus low SNAS (4.26) was different to our initial study, decision tree analysis confirmed a threshold of 5 for CNR in this study also, thus validating our initial analysis in a second cohort using an identical threshold for survival analysis. High CNR was also associated with a number of good prognostic features including ER positivity and low grade (Table 1). Conversely, a high SNAS was associated with high grade, Ki-67 positive tumours (Table 1), suggesting that nuclear survivin is associated with a proliferative phenotype.

These data further validate the hypothesis that the nuclear and cytoplasmic fractions of survivin have different biological roles [23] and support an important role for nuclear-cytoplasmic transport of survivin in tumourigenesis and disease progression [24,25]. Nucleo-cytoplasmic shuttling of survivin is controlled by an evolutionary conserved Crm1-dependent nuclear export signal (NES). A number of groups have demonstrated that inhibition of this signal abrogates the anti-apoptotic effect of survivin, while maintaining its mitotic effect activity, suggesting that increased levels of nuclear survivin could lead to a proliferative aggressive phenotype [24,26,27].

As mentioned previously, the prognostic relevance of survivin in breast cancer is a controversial issue and a number of smaller qualitative IHC-based studies have produced conflicting results. It is possible that the quantitative measurement of survivin (either by ELISA or image analysis) is necessary for its utilisation as a breast cancer biomarker. Interestingly, our initial study [15], as well as those of Span *et al. *[28] and Ryan *et al. *[11], used quantitative methods to evaluate survivin expression and found similar results. The added benefit of our approach is that formalin-fixed paraffin-embedded materials, as opposed to frozen tissue specimens, can be used.

In this study, we were also able to perform subset analysis and demonstrate that a low CNR predicts poor outcome in tamoxifen-treated patients (Table 3). A number of groups have demonstrated that survivin is associated with tamoxifen resistance *in vitro *[29,30]. Span *et al *[28] reported that increased levels of survivin expression (quantified using ELISA) were associated with a good response to chemotherapy, but a poor response to endocrine therapy. We were unable to examine response to chemotherapy in this study, as only 23 patients received adjuvant systemic chemotherapy. Here, survivin expression was examined in 89 tamoxifen-treated patients, which compares adequately with Span *et al *who examined survivin expression in 73 patients treated with tamoxifen [28] and adds further evidence that survivin may play an important role in anti-endocrine resistance. It should be acknowledged that these patients did not participate in a prospective randomised trial, and the predictive value of survivin CNR in tamoxifen-treated patients should be validated in such a setting.

These data add further evidence to the theory that inhibition of survivin may be a viable therapeutic option. A number of phase I and II trials evaluating small molecule inhibitors, antisense nucleotides and immunotherapy targeted against survivin are ongoing [31]. Our data suggest that inhibition of nuclear, as opposed to cytoplasmic, survivin will render the best results, and that the combination of anti-survivin therapies and tamoxifen may be an attractive therapeutic option in a subgroup of ER-positive patients. Further studies will be required to shed light on the value of nuclear and cytoplasmic survivin expression as a surrogate markers of response to any new treatment, with the image analysis solution presented here potentially providing important information in this regard.

## Conclusion

In conclusion, we have validated our previously published method of quantitatively determining the expression of survivin via IHC. Our data add further evidence to the hypothesis that the different sub-cellular pools of survivin have distinct functions and increased levels of nuclear survivin are associated with a proliferative phenotype. Additionally, we have demonstrated that nuclear survivin may predict outcome for tamoxifen-treated breast cancer patients. However, this will require further validation in a prospective cohort. The quantitative image analysis approach described here may be helpful in further dissecting the debate surrounding the role of survivin IHC as a prognostic marker in breast cancer and may be particularly beneficial if ongoing trials of anti-survivin therapies are successful.

## Abbreviations

BCSS: breast cancer specific survival; CI: confidence interval; CNR: Cytoplasmic-to-Nuclear ratio; ER: estrogen receptor; HR: hazard ratio; IHC: immunohistochemistry; NES: nuclear export signal; OS: overall survival; PR: progesterone receptor; qRT-PCR: quantitative real time polymerase chain reaction; SAGE: serial analysis of gene expression; SNAS: Survivin Nuclear Autoscore

## Competing interests

W. Gallagher, E. Rexhepaj and D. Brennan are co-inventors of a pending patent application surrounding an automated image analysis approach, which forms a key basis of the commercial image analysis product, *IHC-MARK*. W. Gallagher is Chief Scientific Officer at OncoMark Limited, which sells *IHC-MARK*.

## Authors' contributions

ER performed image analysis, statistical analysis and drafted the manuscript, KJ constructed the TMAs, DPO'C conceived the study and drafted the manuscript, SLO'B performed IHC and conceived the study, GL constructed the TMAs, MJD conceived the study and drafted the manuscript, DJB conceived the study, performed statistical analysis and drafted the manuscript, WMG conceived the study and drafted the manuscript. All authors read and approved the final manuscript.

## Author information

ER is a bioinformatician, KJ is a molecular pathologist, DPO'C and SLO'B are molecular biologists, GL is a molecular pathologists, MJD is a clinical biochemist, DJB is a clinician scientist, WMG is a cell and molecular biologist.

## Pre-publication history

The pre-publication history for this paper can be accessed here:

http://www.biomedcentral.com/1471-2407/10/639/prepub

## Supplementary Material

Additional file 1 **Training and validation of survivin pattern recognition algorithm**. **(A) **Representative areas from tumour positive, tumour negative, tumour stroma, and slide background were selected as a training set to develop a pattern recognition algorithm. **(B) **Each area was labelled according to the pattern it represents, to provide the evolutionary pattern recognition algorithm with ground truth data to calculate performance of candidate solutions, with the following colour coding being used: tumour positive = red, tumour negative = blue, tumour stroma = green, slide background = cyan. **(C) **Representative areas from tumour positive, tumour negative, tumour stroma and slide background were selected as a validation set to develop a pattern recognition algorithm. **(D) **Each area was labelled according to the pattern it represents to provide ground truth data to the calculate performance of the final solution for recognition of the following: tumour = red, tumour stroma = green, slide background = yellow. Click here for file

Additional file 2 **Automated quantification of low and high scores for SNAS and CNR**. Original IHC images of representative tissue cores with low and high CRN and SNAS patterns. Corresponding automated output from Genie pattern recognition algorithm is shown in second column. Click here for file
