# Supplementary figures and images for: Validation of cytoplasmic-to-nuclear ratio of survivin as an indicator of improved prognosis in breast cancer
Source: BMC Cancer. 2010 Nov 23;10:639. doi: 10.1186/1471-2407-10-639 (PMC2999619; doi:10.1186/1471-2407-10-639)

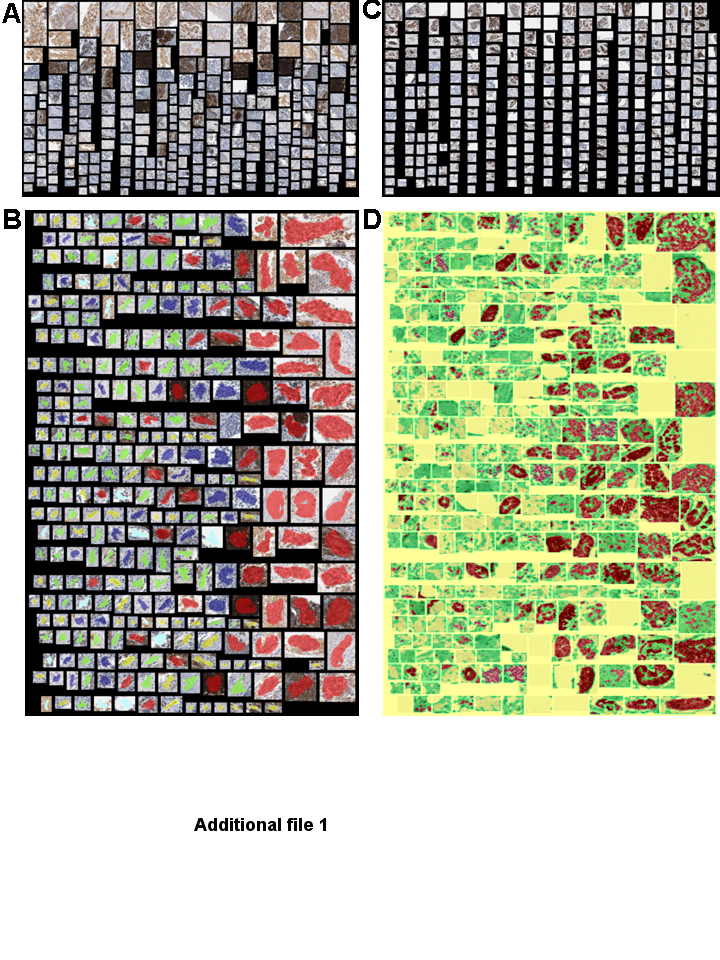

Supplement: Additional file 1 — Training and validation of survivin pattern recognition algorithm. (A) Representative areas from tumour positive, tumour negative, tumour stroma, and slide background were selected as a training set to develop a pattern recognition algorithm. (B) Each area was labelled according to the pattern it represents, to provide the evolutionary pattern recognition algorithm with ground truth data to calculate performance of candidate solutions, with the following colour coding being used: tumour positive = red, tumour negative = blue, tumour stroma = green, slide background = cyan. (C) Representative areas from tumour positive, tumour negative, tumour stroma and slide background were selected as a validation set to develop a pattern recognition algorithm. (D) Each area was labelled according to the pattern it represents to provide ground truth data to the calculate performance of the final solution for recognition of the following: tumour = red, tumour stroma = green, slide background = yellow. [file 1471-2407-10-639-S1.TIFF]

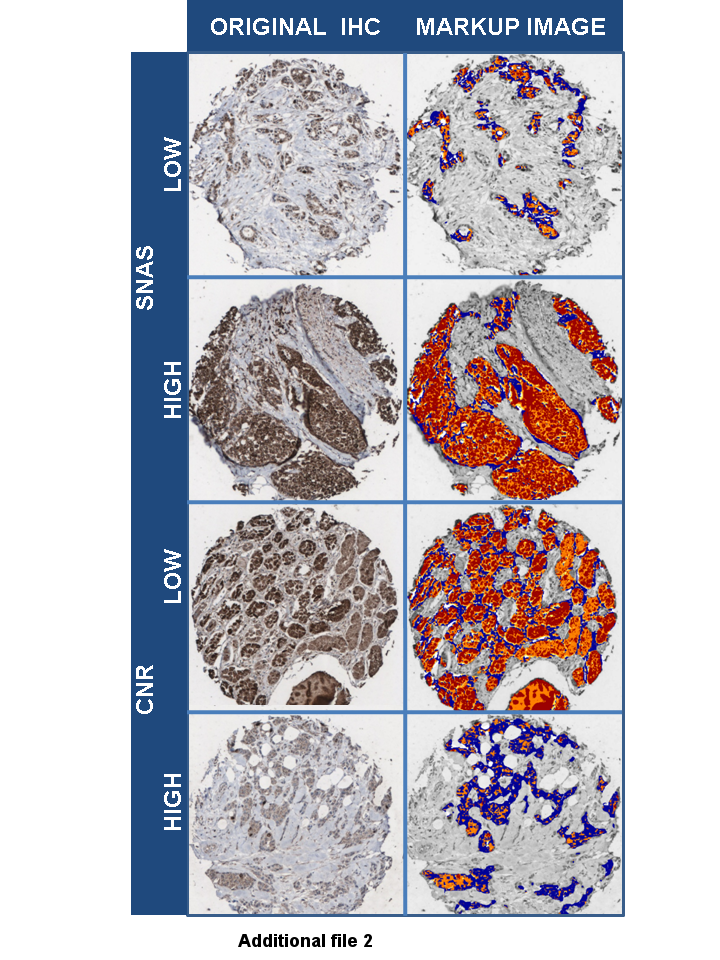

Supplement: Additional file 2 — Automated quantification of low and high scores for SNAS and CNR. Original IHC images of representative tissue cores with low and high CRN and SNAS patterns. Corresponding automated output from Genie pattern recognition algorithm is shown in second column. [file 1471-2407-10-639-S2.TIFF]
